# Transglutaminase 2: a novel therapeutic target for idiopathic pulmonary fibrosis using selective small molecule inhibitors

**DOI:** 10.1007/s00726-020-02938-w

**Published:** 2021-01-21

**Authors:** Shaun Fell, Zhuo Wang, Andy Blanchard, Carmel Nanthakumar, Martin Griffin

**Affiliations:** 1 School of Life and Health Sciences, Aston University, Birmingham, UK; 2 Fibrosis Discovery Performance Unit, Respiratory Therapy Area, Medicines Research Centre, GlaxoSmithKline R and D, Stevenage, UK

**Keywords:** Transglutaminase 2, Idiopathic pulmonary fibrosis, Transforming growth factor β1, Myofibroblasts

## Abstract

This study investigates the effects of a site-directed TG2-selective inhibitor on the lung myofibroblast phenotype and ECM deposition to elucidate TG2 as a novel therapeutic target in idiopathic pulmonary fibrosis (IPF)—an incurable progressive fibrotic disease. IPF fibroblasts showed increased expression of TG2, α smooth muscle actin (αSMA) and fibronectin (FN) with increased extracellular TG2 and transforming growth factor β1 (TGFβ1) compared to normal human lung fibroblasts (NHLFs) which do not express αSMA and express lower levels of FN. The myofibroblast phenotype shown by IPF fibroblasts could be reversed by selective TG2 inhibition with a reduction in matrix FN and TGFβ1 deposition. TG2 transduction or TGFβ1 treatment of NHLFs led to a comparable phenotype to that of IPF fibroblasts which was reversible following selective TG2 inhibition. Addition of exogenous TG2 to NHLFs also induced the myofibroblast phenotype by a mechanism involving TGFβ1 activation which could be ameliorated by selective TG2 inhibition. SMAD3-deleted IPF fibroblasts via CRISPR-cas9 genome editing, showed reduced TG2 protein levels following TGFβ1 stimulation. This study demonstrates a key role for TG2 in the induction of the myofibroblast phenotype and shows the potential for TG2-selective inhibitors as therapeutic agents for the treatment of fibrotic lung diseases like IPF.

## Introduction

Idiopathic pulmonary fibrosis (IPF) is an incurable and life-threatening disease with a median survival rate of 2–3 years in the UK (Strongman et al. 2018). The most common cause of death is respiratory failure, accounting for over 80% of deaths (Raghu et al. 2006). Histological analysis of IPF samples show thick collagen and ‘fibroblast foci’ in areas of fibrosis (Kekevian et al. 2014). Increased proliferation of fibroblasts and excessive deposition of extracellular matrix (ECM) proteins above the rate of ECM degradation characterises IPF (Dragsbaek et al. 2015; Ramos et al. 2001). It has been demonstrated by Mora et al. that there has been a high attrition rate for drugs that enter clinical trials for the treatment of IPF, and, therefore, this highlights the demand for promising new targets to be identified (Mora et al. 2017).

Transglutaminase 2 (TG2), is a calcium-activated multifunctional enzyme, well known for the formation of ε-(γ-glutamyl)lysine isopeptide bonds that lead to protein cross-linking, that provide resistance to proteolytic cleavage and ultimately, stabilisation and increased stiffness of the ECM (Bergamini et al. 2011; Wang and Griffin 2012). TG2 is reported to have various pathological roles, including cancer metastases (Ayinde et al. 2017; Kotsakis et al. 2011; Wang and Griffin 2013), coeliac disease (Nadalutti et al. 2013) and tissue fibrosis (Badarau et al. 2015; Wang et al. 2018). TG2 knockout (KO) mice show reduced fibrosis in response to bleomycin-induced pulmonary fibrosis, including a marked reduction in collagen deposition (Oh et al. 2011). However, the actual mechanism(s) of TG2′s role in IPF remains unknown. Importantly, TG2-selective inhibitors have not yet been tested in IPF.

Use of irreversible small molecule TG2 inhibitors in a rat diabetic nephropathy model showed that TG2 inhibition led to reduced glomerulosclerosis and tubulointerstitial fibrosis by up to 77% and 92%, respectively (Johnson et al. 2007). Recently, the TG2-selective and irreversible inhibitor 1–155 used in this study and developed at Aston University resulted in upto a 40% reduction in collagen deposition in a mouse Angiotensin II model of nephrosclerosis and a 60% reduction in infarct size in an acute myocardial infarction mouse model (Badarau et al. 2015; Wang et al. 2018). These previous studies demonstrate the potential of site-directed TG2-selective inhibitors for the treatment of tissue fibrosis.

Apart from its importance in generating a protease-resistant crosslinked fibrotic matrix, TG2 can release the active transforming growth factor β1 (TGFβ1) from ECM via crosslinking the large latent TGFβ1-binding protein (LTBP)-TGFβ1 complex in the ECM (Verderio et al. 1999). TGFβ1 is a crucial pro-fibrotic cytokine that promotes fibrosis via fibroblast-to-myofibroblast transition (Wang et al. 2018), epithelial–mesenchymal transition (EMT) (Nyabam et al. 2016) and endothelial–mesenchymal transition (EndMT) (Wang et al. 2017), promoting myofibroblast activation, inducing ECM protein expression and deposition and inhibiting matrix degradation pathways leading to matrix accumulation.

Here we demonstrate how TG2 is involved in inducing the myofibroblast phenotype in IPF cells. We also demonstrate the potential of using site-directed TG2-selective inhibitors as therapeutic agents for IPF. We show that these inhibitors can reduce matrix deposition and reverse the myofibroblast phenotype in IPF via preventing the activation of TGFβ1, reducing myofibroblast biomarkers and inhibiting excessive matrix protein deposition.

## Results

### Increased expression of TG2 and myofibroblast markers in IPF fibroblasts

**Fig. 1:**
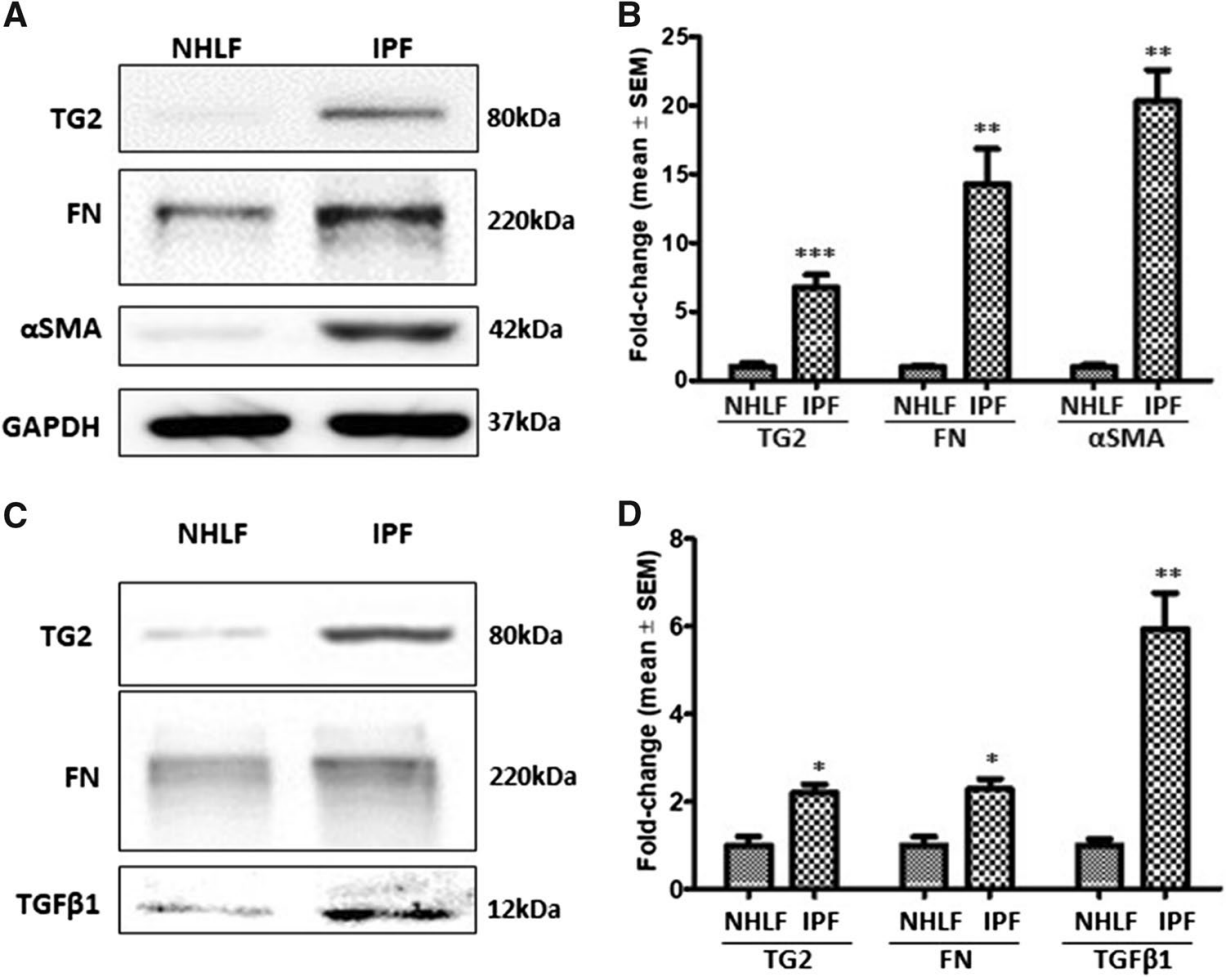
Characterisation of NHLFs and IPF fibroblasts. **a** Representative Western blots of TG2, FN and αSMA in the cell lysates of NHLFs and IPF fibroblasts. GAPDH was used to normalise loading. **c** Representative blots of TG2, FN and TGFβ1 in ECM fractions of NHLFs and IPF fibroblasts. **b** and **d** are graphs showing mean densitometry values ± S.D. with the NHLFs taken as 1.0. *, *p* < 0.05; **, *p* < 0.01; and ***, *p* < 0.001

Previous research has identified that IPF is associated with an increase in the myofibroblast presence and excessive ECM deposition (Olsen et al. 2011). TG2 was significantly upregulated in IPF fibroblasts, in comparison to NHLFs (Fig. 1a and b) with IPF fibroblasts showing a significant increase in both FN and α smooth muscle actin (αSMA), compared to NHLFs. Increased levels of TG2, fibronectin (FN) and TGFβ1 were also detected in the ECM fractions deposited by IPF fibroblasts when compared to NHLFs (Fig. 1c and d).

### Overexpression of active TG2 leads to a myofibroblast phenotype in NHLFs

**Fig. 2:**
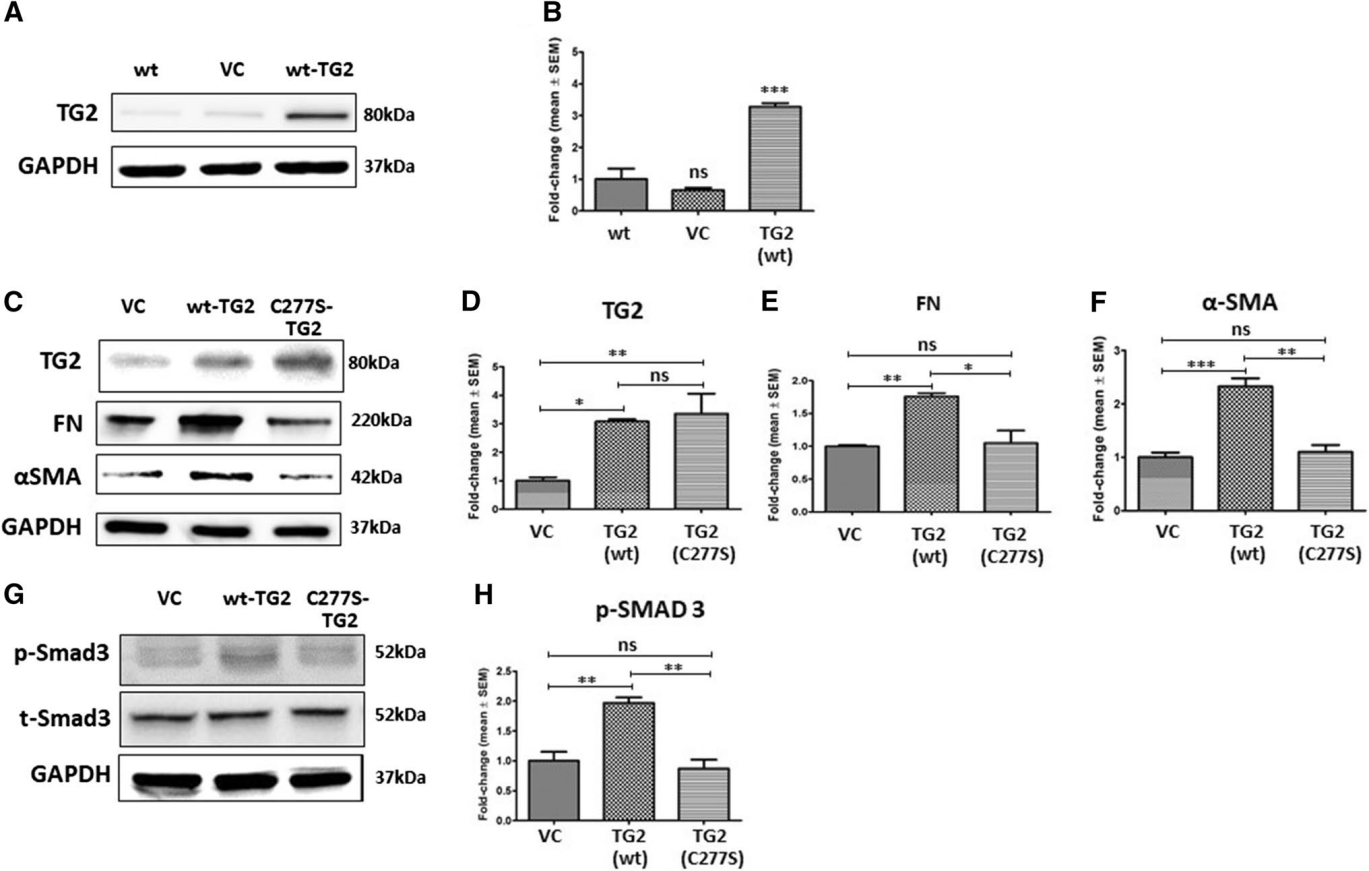
Overexpression of catalytically active TG2 in NHLFs induces Smad3 signalling and a myofibroblast phenotype. **a** Representative Western blot of TG2 in NHLFs cell lysates following lentiviral transduction. VC, vector control. **b**, **c** Representative Western blots of TG2, FN and αSMA in NHLFs transduced with empty vector (VC), wt-TG2 and C277S-mutant TG2 (C277S-TG2). **d**–**g** Representative Western blots of p-Smad3 in VC, wt-TG2 and C277S-TG2 lentivirus transduced NHLFs. Graphs based on densitometry analysis of Western blots are shown in **b**–**f** and **h** expressed as a mean fold change ± SEM compared with VC or NHLFs control taken as 1.0. GAPDH was used to ensure equal loading. *, *p* < 0.05; **, *p* < 0.01; ***, *p* < 0.001; and NS, not significant

Wild-type (wt) TG2 overexpression in NHLFs via lentiviral transduction gave rise to increased TG2 protein levels in comparison to non-transduced or control virus-transduced NHLFs (Fig. 2a and b). A transamidating-inactive mutant TG2 (C277S-TG2) (Balklava et al. 2002) was also overexpressed in NHLFs to understand the importance of the transamidating activity of TG2 (Fig. 2c–f). Western blotting showed significant increases in TG2 expression confirming successful transduction of both wild type and C277S mutant TG2 compared to control vector transduced and wild type NHLFs. As expected, both FN and αSMA expression were increased in wild-type TG2-transduced NHLFs; however, no increases in FN and αSMA were detected in the inactive C277S-TG2 trarnsduced NHLFs (Fig. 2c–f). Increases in Smad3 phosphorylation (p-Smad3) was only detected in the wild type active TG2 transduced NHLFs when compared to the vector control and C277S mutant transduced cells (Fig. 2g and h) confirming the importance of active TG2 in myofibroblast formation.

### TGFβ1 involvement in the TG2-regulated myofibroblast transition in NHLFs

**Fig. 3:**
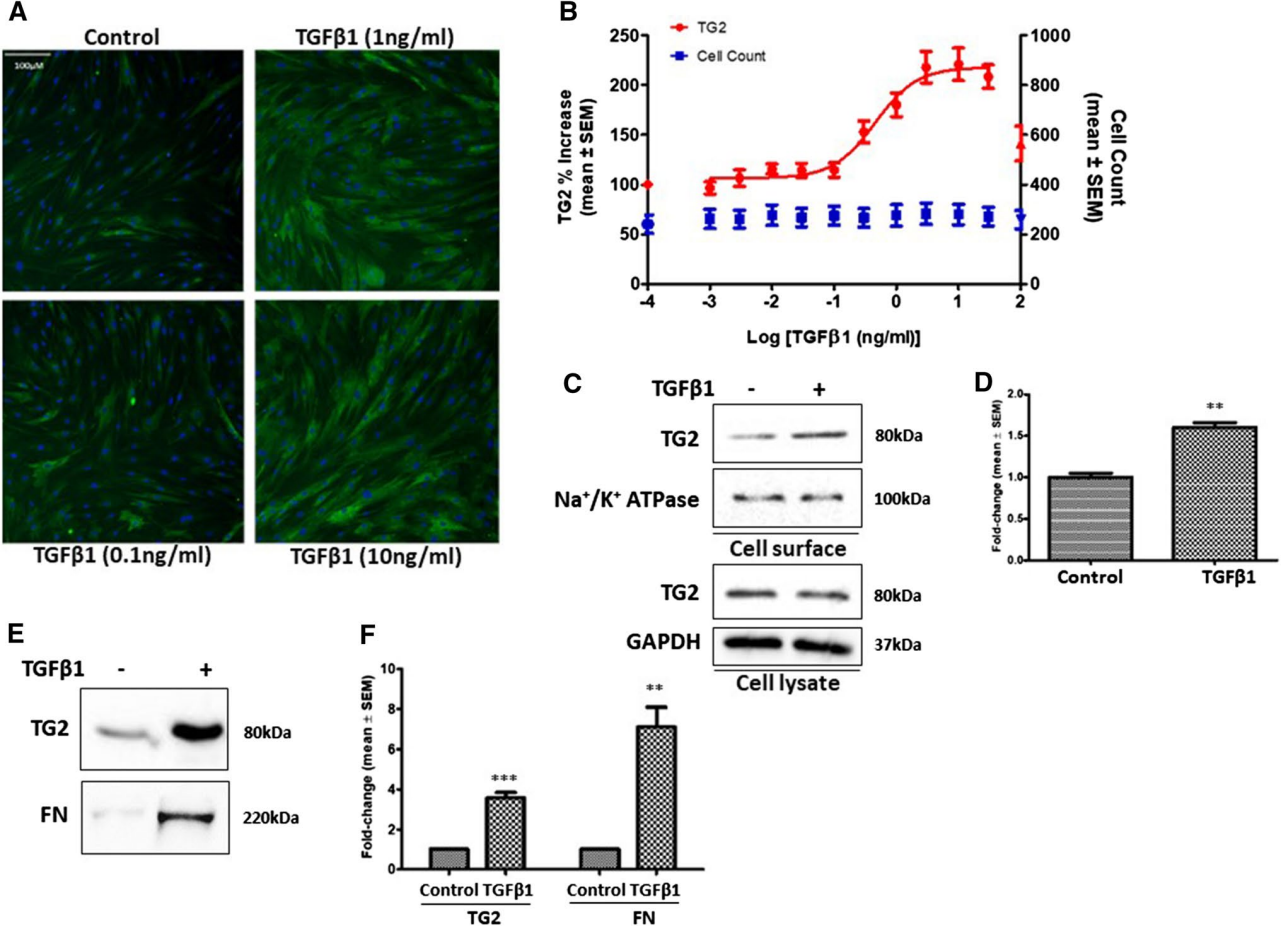
TGFβ1 stimulation of NHLFs regulates TG2 protein levels and ECM deposition. **a** Representative images of TG2 staining (green) in NHLFs treated with different concentrations of TGFβ1 with Hoechst (blue) staining. **b** Dose–response curve of TG2 levels against log TGFβ1 concentration. Mean percentage increase ± SEM in cell count (blue) also shown. The images were quantified as described in “Materials and methods”. **c**–**f** Representative Western blots of TG2 in cell surface and cell lysates **c** and matrix TG2 and FN **f** following treatment of NHLFs with TGFβ1 (1 ng/ml). Na + /K + ATPase was used as a cell surface loading control. Graphs based on densitometry analysis of Western blots with controls normalised to 1.0 are shown in **d** and **f**. GAPDH was used to ensure equal loading for cell lysates. Data are shown as a mean fold change ± SEM of control cells. **, *p* < 0.01; and ***, *p* < 0.001

Our previous work suggested that TGFβ1, a pro-fibrotic cytokine known to increase during fibrosis can upregulate TG2 expression (Nyabam et al. 2016; Wang et al. 2018). Fluorescence staining of TG2 in NHLFs confirmed increased TG2 expression following stimulation of NHLFs with TGFβ1 (Fig. 3a and b).Cell surface biotinylation revealed higher levels of cell surface TG2, confirmed using Na + /K + ATPase as a cell surface marker, in TGFβ1-treated NHLFs (Fig. 3c and d), while a significant increase in ECM TG2 was also found in these cells (Fig. 3e and f), accompanied with increased FN deposition (Fig. 3e and f).

### Exogenous active TG2 leads to a myofibroblast phenotype in NHLFs via TGFβ1

**Fig. 4:**
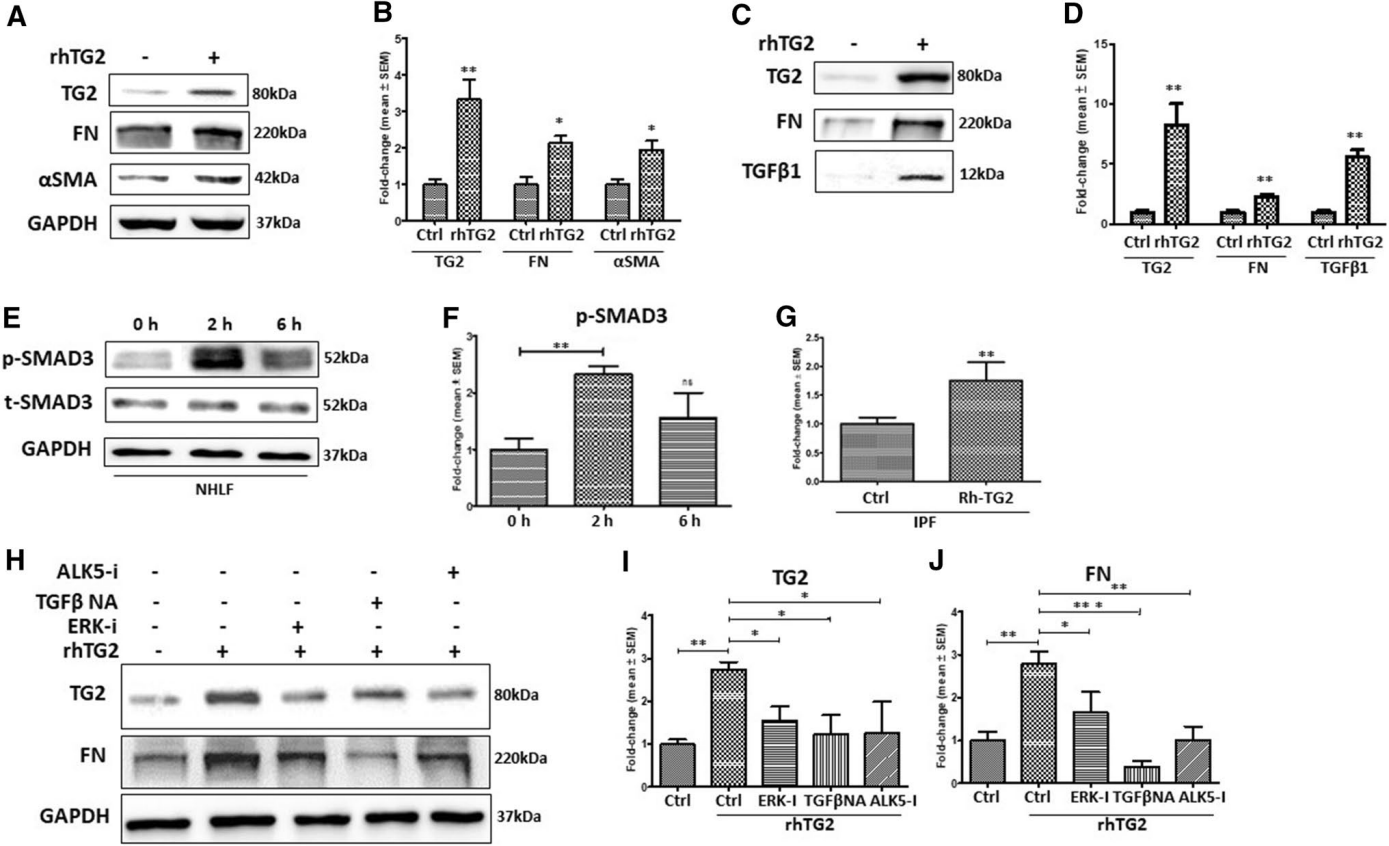
The effect of Rh-TG2 stimulation on NHLFs lysate and matrix protein levels. **a** Representative Western blots of TG2, FN and αSMA and **c** matrix TG2, FN and monomeric TGFβ1 in NHLFs treated with exogenous Rh-TG2 (1 µg/ml) and untreated NHLFs. **e** Representative Western blots of p-SMAD3 and t-SMAD3 in control NHLFs and NHLFs treated with 1 µg /ml Rh-TG2 for 2 or 6 h. **g** Graph showing the presence of active TGFβ in IPF fibroblasts treated with Rh-TG2 (1 µg/ml) via the Mink TGFβ reporter system as described in the “Materials and methods”. **h** Representative Western blots of TG2 and FN in NHLFs treated with an ERK inhibitor (10 µM), a TGFβ neutralising antibody (20 µg/ml) or an Alk5 inhibitor (10 µM) in the presence of Rh-TG2 (1 µg/ml). Graphs based on densitometry analysis of Western blots with controls normalised to 1.0 are shown in **b**–**i** and **j** expressed as a mean fold change ± SEM. GAPDH was used to ensure equal loading. *, *p* < 0.05; **, *p* < 0.01; and ***, *p* < 0.001

Given that TG2 and TGFβ1 can form a positive feedback cycle in driving myofibroblast transition, including cystic and cardiac fibrosis (Nyabam et al. 2016; Wang et al. 2018), we next studied whether addition of exogenous TG2 to NHLFs can lead to their transition into myofibroblasts. Figure 4a and b shows that the addition of exogenous rh-TG2 resulted in an increase in TG2 protein levels in NHLFs cell lysates. Importantly, exogenous TG2 treatment also increased FN and αSMA expression, compared to untreated NHLFs (Fig. 4a and b), which was accompanied by higher levels of ECM TG2, FN and TGFβ1 (Fig. 4c and d). Compared to the control NHLFs, introduction of Rh-TG2 to NHLFs triggered the activation of SMAD3 (p-SMAD3) within 2 h to around 2.5 times the control which subsequently declined to around 1.5 times the control NHLFs value after 6 h (Fig. 4e and f). This suggests that exogenous TG2 is acting via induction of TGFβ signalling which is supported by the finding of a 70% increase of active TGFβ in the culture medium of TG2 treated IPF cells measured using the Mink lung epithelial cells (MLECs) reporter system (Fig. 4g) and the presence of increased active TGFβ in the matrix of TG2 treated NHLFs (Fig. 4c).

In addition, treatment of NHLFs cells with inhibitors of ERK signalling, ALK5 receptor or a TGFβ neutralising antibody (TGFβ-NA) all resulted in the inhibition of the increased expression of TG2, FN and αSMA induced by exogenous TG2 (Fig. 4h–j), confirming that exogenous TG2 functions through the TGFβ1 pathway(s) in inducing TG2, FN and increased TGFβ in NHLFs.

### Selective inhibition of TG2 reverses the TGFβ1-induced myofibroblast phenotype in NHLFs

**Fig. 5:**
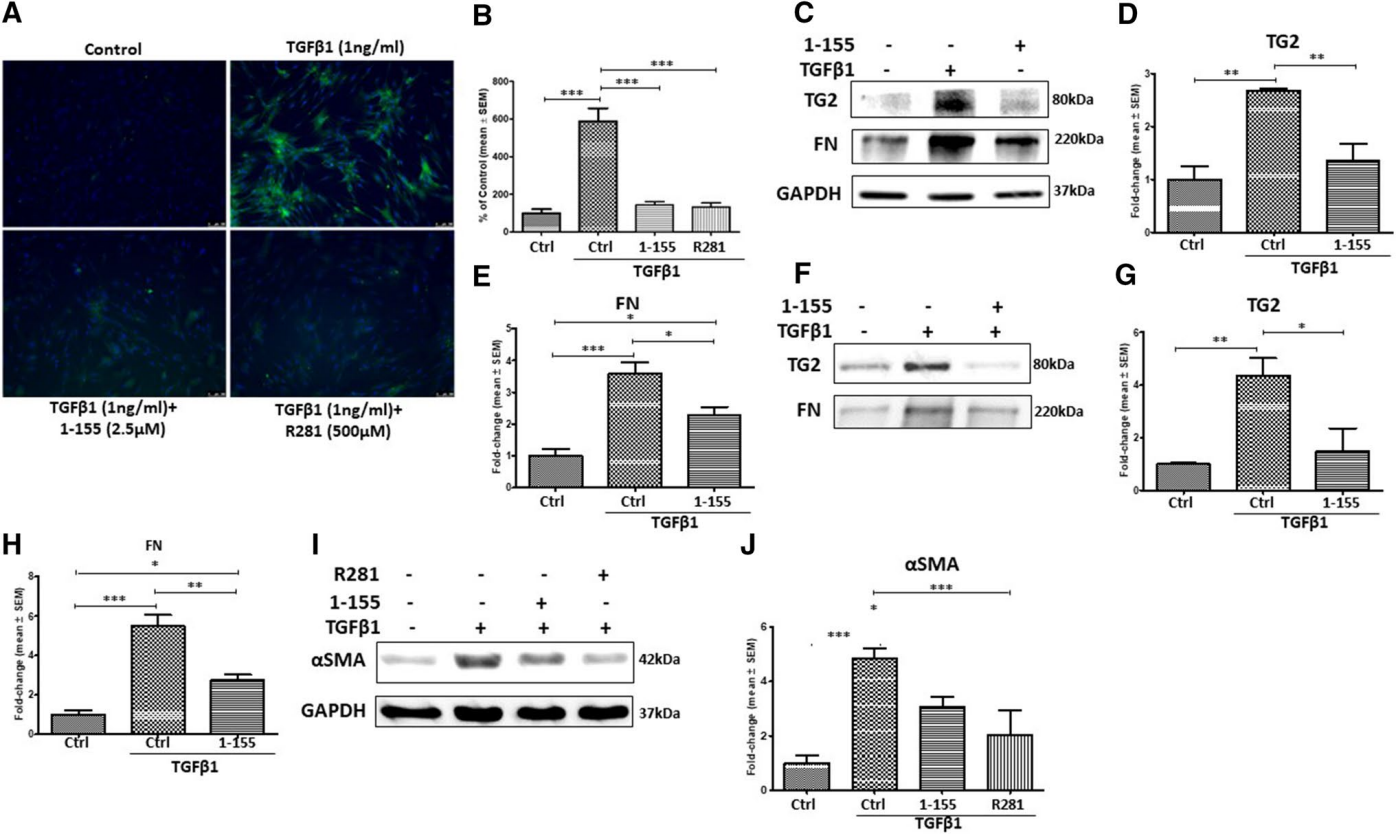
TG2-selective inhibitors prevent the TGFβ1-induced increases in TG2 activity, the myofibroblast phenotype and ECM protein deposition. **a** TG2 in situ activity measured via FITC-cadaverine incorporation following treatment of NHLFs with TGFβ1 (1 ng/ml) and TG2 inhibitors (1–155 and 281). TG2 activity (green) and DAPI (blue). **b** Relative fluorescent signals of treated NHLFs against the control. Control fluorescent signals were normalized to 100%. Fluorescent signals were measured via ImageJ software. **c** Representative Western blots of TG2 and FN in control and NHLFs treated with TGFβ1 and TG2 inhibitor 1–155. **f** Representative Western blots of TG2 and FN from ECM fractions of NHLFs treated with TGFβ1 and TG2 inhibitor 1–155. **i** Representative Western blots of αSMA in control NHLFs, TGFβ1-treated NHLFs and NHLFs treated with TG2 inhibitor 1–155 or R281 with TGFβ1. Graphs based on densitometry analysis of Western blots with controls normalised to 1.0 are shown in **b**–**h** and **j**. GAPDH was used to ensure equal loading data in the graphs are expressed as a mean fold change ± SEM compared to the control NHLFs. *, *p* < 0.05; **, *p* < 0.01; and ***, *p* < 0.001

Selective inhibitors of TG2 can reduce heart and kidney fibrosis (Badarau et al. 2015; Wang et al. 2018); however, this has not been tested in IPF. Figure 5a and b show that TG2-selective inhibitor 1–155 and a non-cell permeable TG2 inhibitor R281 were able to significantly reduce the in situ TG2 activity in NHLFs induced by TGFβ1 treatment. Similarly, 1–155 treatment decreased the TGFβ1-induced expression of TG2 and FN (Fig. 5c–e) and reduced matrix deposition (Fig. 5f–h) of TG2 and FN. Importantly, 1–155 and R281 treatment was able to block the TGFβ1-induced myofibroblast marker αSMA in NHLFs (Fig. 5i and j).

### TGFβ1-mediated TG2 expression involves Smad3

**Fig. 6:**
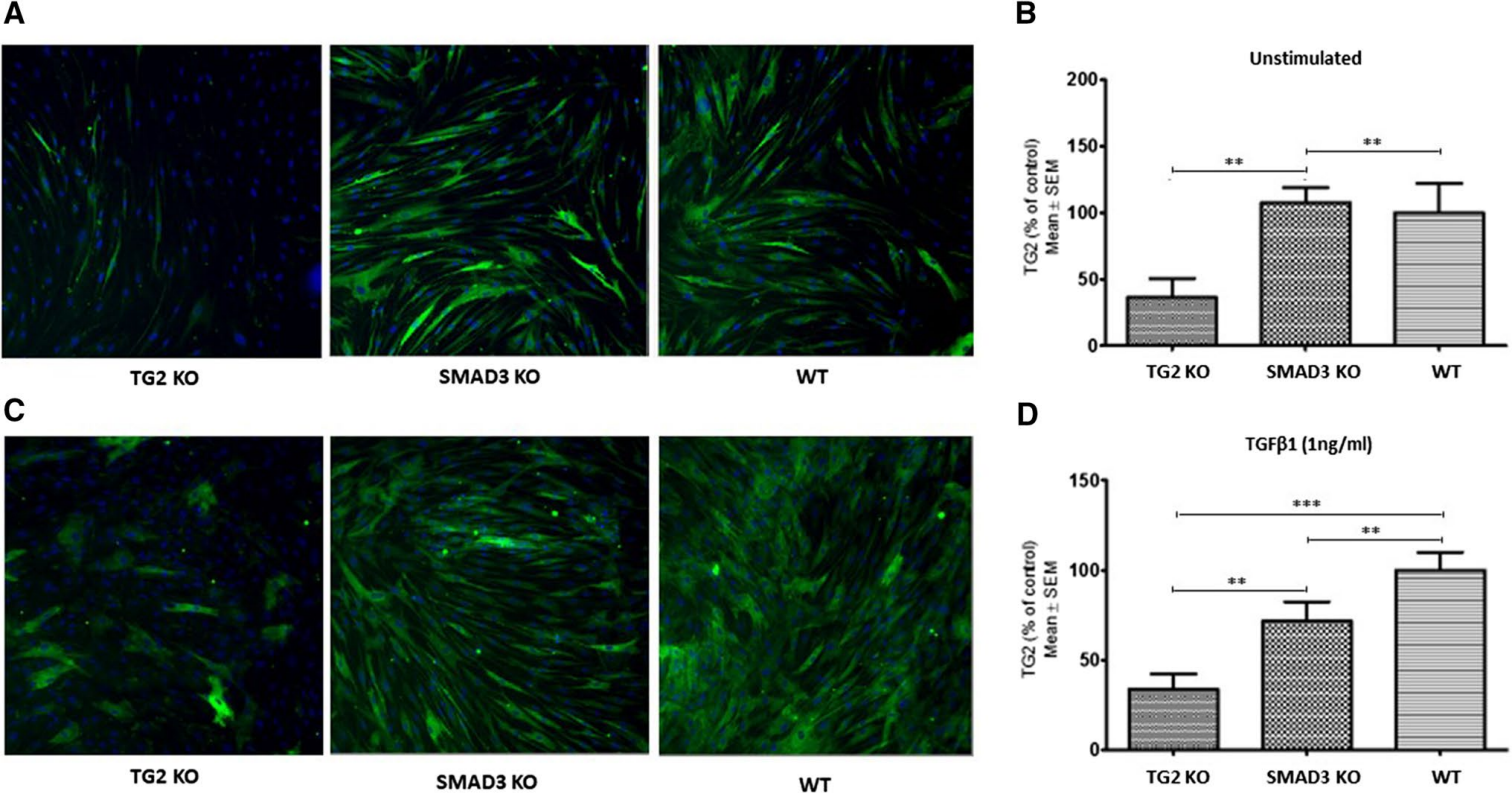
CRISPR-Cas9 genome editing was performed on IPF fibroblasts targeting the Smad3 and TGM2 gene. IPF fibroblasts underwent CRISPR-Cas9 genome editing as described in the “Materials and methods” targeting the SMAD3 and TGM2 genes. Representative images of immunocytochemistry staining of TG2 in the TG2 KO, SMAD3 KO and WT IPF fibroblasts in the absence **a** and presence **c** of TGFβ1 stimulation. TG2 (green), Hoechst (blue). **b** and **d** Graphs present the percentage of the signals measured via immunocytochemistryas described in the “Materials and methods”. Quantitative data was generated using the ‘Mean_TargetAvgIntenCh2′ algorithm and the ‘AP30704v1_SIAJCollagenandCellCountCellHealth (V01)’ protocol. Data are expressed as a percentage of control ± SEM. **, *p* < 0.01; and ***, *p* < 0.001

TG2 expression has been reported to be regulated via the non-canonical TGFβ1 pathway through the activation of ERK1/2 in NHLFs (Olsen et al. 2011), but the role of the TGFβ1 canonical pathway via SMADs has not been studied. Our data so far suggest that the involvement of TGFβ1 canonical pathway via Smad3 is involved in TG2-mediated myofibroblast transition in NHLFs and IPF fibroblasts. To confirm this, we used CRISPR-Cas9 genome editing to knockout Smad3 protein expression (SMAD3-KO) and TG2 expression (TG2–KO) in IPF fibroblasts. TG2 expression was then measured via immunofluorescence staining in SMAD3-KO and TG2-KO cells both in the absence (Fig. 6a and b) and presence of TGFβ1 stimulation (Fig. 6c and d). TG2-KO IPF fibroblasts which had around 65% of cells with TG2 gene editing, failed to respond to TGFβ1 stimulation. Smad3-KO IPF fibroblasts which had around 60% of cells showing the absence of Smad3 expression showed no significant change in TG2 expression compared to WT IPF fibroblasts in unstimulated conditions (Fig. 6a and b). However, SMAD3-KO IPF fibroblasts did show a significant decrease (approx. 25%) in TG2 expression following TGFβ1 treatment, compared to wild-type IPF fibroblasts (Fig. 6c and d), confirming the importance of Smad3 in TGFβ1 regulated TG2 expression.

### Selective TG2 inhibition can reverse the myofibroblast phenotype in IPF fibroblasts

**Fig. 7:**
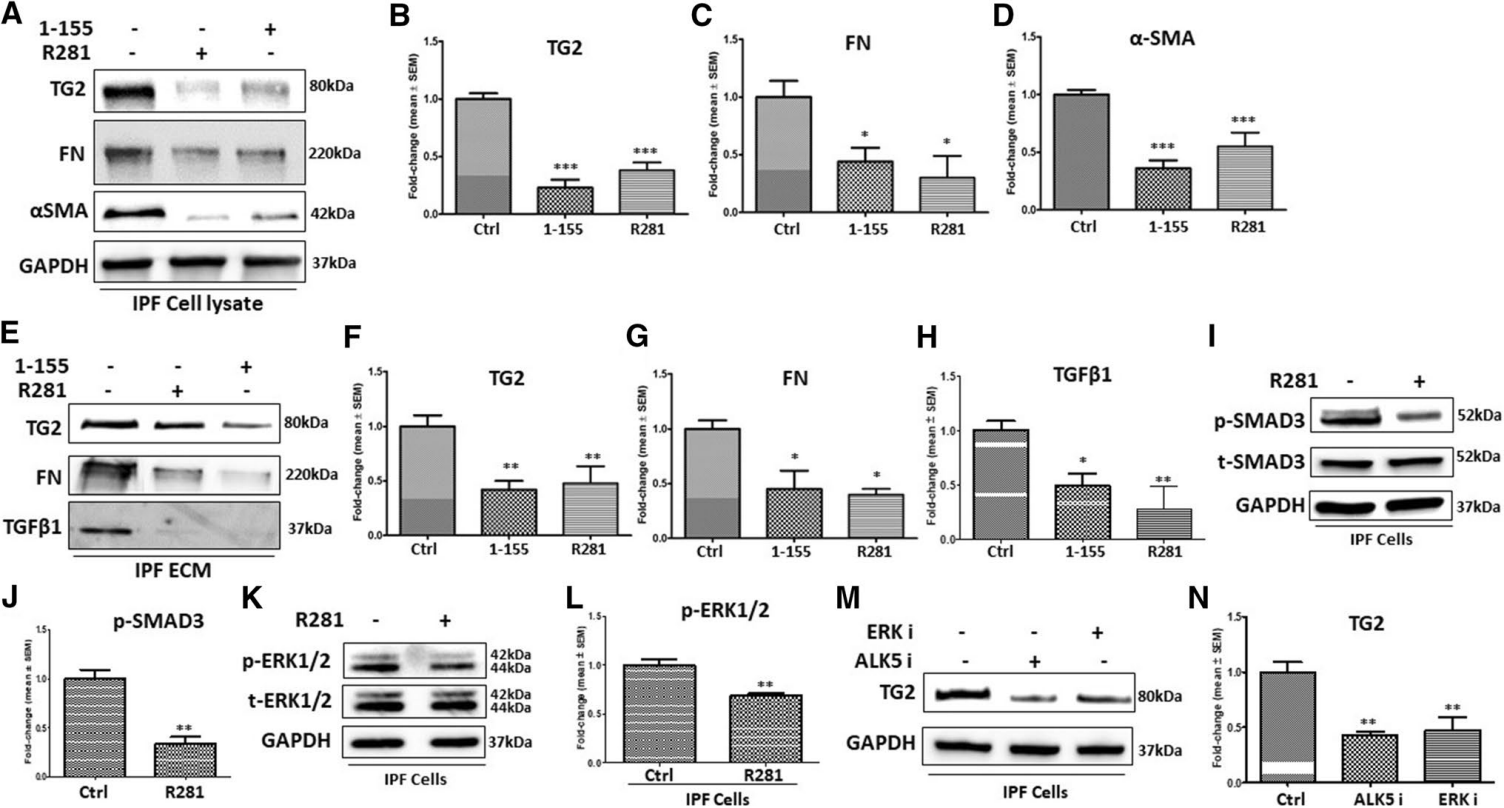
Treatment of IPF fibroblasts with TG2-specific inhibitors and the relationship with Smad3 and ERK1/2 signalling pathways. **a** Representative Western blot of TG2, FN and αSMA in control IPF fibroblasts and IPF fibroblasts treated with either TG2 inhibitor 1–155 (2.5 µM) or R281 (500 µM). **e** Representative Western blots of TG2, FN and TGFβ1 from ECM fractions of IPF cells treated as shown. **i** Representative Western blots of p-SMAD3 and t-SMAD3 and **k** p-ERK1/2 and t-ERK1/2 in the control IPF fibroblasts and R281 treated IPF fibroblasts. **m** Representative Western blots of TG2 in control IPF fibroblasts and IPF fibroblasts treated with an ALK5 or an ERK inhibitor. Graphs based on densitometry analysis of Western blots with controls normalised to 1.0 are shown in **b**–**l** and **n**. GAPDH was used to ensure equal loading data in the graphs are expressed as a mean fold change ± SEM compared to the control NHLFs *, *p* < 0.05; **, *p* < 0.01; and***, *p* < 0.001

We next treated IPF fibroblasts with TG2 inhibitors to explore the potential of selective TG2 inhibition as a therapeutic avenue for IPF. IPF fibroblasts were treated with the extracellular acting TG2 inhibitor R281 (500 µM) and the TG2-selective inhibitor 1–155 (2.5 µM) for 72 h. Figure 7a and b show that both TG2-selective cell-permeable inhibitor 1–155 and cell-impermeable inhibitor R281 reduced TG2 expression in IPF fibroblast lysates, accompanied with significant decreased expression of FN and αSMA (Fig. 7a, c and d) and reduced TG2, FN and monomeric TGFβ1deposition in the ECM (Fig. 7e–h).

Figure 7i and j demonstrate that treatment of IPF fibroblasts with cell impermeable TG2 inhibitor R281 led to a reduction in p-SMAD3 levels compared to the control IPF fibroblasts, with p-ERK1/2 levels also decreasing in the presence of R281 (Fig. 7k and l). To gain a greater understanding of the regulation of TG2 expression, IPF fibroblasts were treated with an inhibitor of ERK and an ALK5 receptor inhibitor. Both ALK5 and ERK inhibition led to a reduction in TG2 expression compared to the control IPF fibroblasts (Fig. 7m and n).

## Discussion

The involvement of TG2 in lung fibrosis was first proposed by (Griffin et al. 1979). Later studies have now shown that increased expression of TG2 is found in the lungs of IPF patients and that TG2-KO mice develop significantly reduced bleomycin-induced fibrosis compared to wild-type mice (Olsen et al. 2014). This same group investigated two small electrophilic compounds CDDO and 15d-PGJ2 that were reported to block myofibroblast formation and matrix deposition in normal human fibroblasts (Olsen et al. 2011). They demonstrated that in normal and IPF-derived fibroblasts that TG2 expression was reduced by these two small molecules although in IPF derived fibroblasts this reduction in TG2 expression was less effective. They concluded that the ability of these small molecules to reduce TG2 expression and inhibit fibrosis was via inhibition of ERK signalling.

More recent studies have demonstrated that the pan transglutaminase inhibitor cystamine reduces collagen deposition in the mouse bleomycin model of lung fibrosis (Philp et al. 2018). However, unlike selective TG2 inhibitors such as 1–155 used in this study, cystamine inhibits other transglutaminases, as well as cysteine proteases, such as Caspase 3.

In this paper, we confirm previous data showing that TG2 protein levels are significantly increased in IPF fibroblasts compared to NHLFs (Olsen et al. 2014). In addition, we demonstrate that treatment of primary IPF fibroblasts with a TG2-selective inhibitor 1–155 and a cell impermeable TG2 inhibitor R281 reverses the myofibroblast phenotype with reduced expression of FN, TG2 and αSMA and reduced deposition into the ECM of TG2, FN and TGFβ1. These data are in keeping with our previous data showing that TGFβ1 induction of cardiofibroblasts into myofibroblasts and the parallel increase in matrix deposition could be inhibited using selective TG2 inhibitor 1–155, which when translated into mouse models of cardiac fibrosis and nephrosclerosis indicated that treatment with 1–155 caused a significant reduction of collagen deposition in these different animal models. Hence it is not unreasonable to assume that TG2 selective inhibitors like 1–155 are able to reduce fibrosis in animal models of lung fibrosis such as the bleomycin model commonly used for preclinical studies.

Our findings confirm previous studies in that over-expression of TG2 in NHLFs leads to increased matrix protein levels (Olsen et al. 2014). In addition, our study also shows that over-expression of TG2 in NHLFs leads to upregulation of αSMA protein levels compared to control cells; however, previous research by Olsen et al. found no differences in αSMA protein (Olsen et al. 2014). NHLFs express very low levels of αSMA, but research by Olsen et al. showed αSMA protein levels to be relatively high in NHLFs transduced with a control virus (Olsen et al. 2014). However, we did not encounter any phenotype changes in empty vector transduced NHLFs. Our study revealed a marked increase in αSMA protein levels following over-expression of TG2 in NHLFs compared to the low levels of αSMA protein in NHLFs transduced with control virus.

TGFβ1 has previously been reported to upregulate TG2 expression by activating the TGFβ1 response element located within the *TGM2* gene promoter (Nunes et al. 1997) which supports our observations within this study. Furthermore, knockout of SMAD3 in IPF cells by CRISPR-Cas9 genome editing suggests that TGFβ1 also utilises SMAD3 signalling for the upregulation of TG2. However, our data also show that SMAD3 is not the sole regulator of TGFβ1-induced upregulation of TG2. Previous research identified a phosphorylation signalling cascade involving ERK activation also regulates TG2 expression and targeting ERK with inhibitors could reduce TG2 expression in fibrotic lung fibroblasts (Olsen et al. 2011). This suggests that TGFβ1-induced upregulation of TG2 is likely to be regulated by both canonical (SMAD3) and non-canonical (ERK) TGFβ1-induced signalling which may explain why 60% knockout of SMAD 3in IPF fibroblasts only gave rise to around 25% decrease in TG2 expression when compared to control cells.

We show that addition of exogenous TG2 to NHLFs leads to increased expression of TG2, FN and αSMA, with increased matrix deposition of TG2, FN and activated TGFβ1. We also show that addition of TG2 to IPF cells which already show the myofibroblast phenotype leads to a significant increase in active TGFβ1 levels in the culture medium. Since TG2 inhibition blocked this response in NHLFs, this suggests that the increased TGFβ1 levels are resulting from TG2 activation of latent matrix-bound TGFβ1,through a mechanism demonstrated by (Nunes et al. 1997).This in turn leads to induction of the myofibroblast phenotype since both the cell impermeable inhibitor 281 and the cell permeable inhibitor 1–155 which acts both at the intra-and extracellular level blocked expression of both TG2 and the myofibroblast marker αSMA. It has also been reported that TG2 crosslinking of the ECM can lock active TGFβ1 into the matrix which may be a mechanism for prolonging the half-life and creating an active reservoir of this transient growth factor (Nyabam et al. 2016; Wang et al. 2017). This phenomenon has also been observed with VEGF (Wang et al. 2013). Further support for such a mechanism comes from the finding that when TGFβ is incorporated into TG crosslinked collagen it remains active and capable of cell signalling (Niger et al. 2013). Addition of exogenous TG2 also led to an increase in activated TGFβ1 presence in the ECM, which supports this theory. Importantly in relation to in vivo studies (Johnson et al. 2007), TG2 has been shown to be found in high levels in the extracellular matrix where it will activate matrix bound latent TGFβ1 leading to a positive feedback-loop between TG2 and TGFβ1 which we demonstrate in primary NHLFs. Targeting TG2 using TG2-selective inhibitors can disrupt this pro-fibrotic feedback loop preventing progressive fibrosis from occurring as we demonstrated in both cardiac (Wang et al. 2018) and kidney fibrosis mice models (Badarau et al. 2015; Johnson et al. 2007).

Previous researchers have suggested that TG2 activity is critical for the extracellular crosslinking of ECM proteins that leads to proteolytic-resistant fibrotic lesions (Collighan and Griffin 2009). We demonstrate that treatment of TGFβ1-stimulated NHLFs with a TG2-selective inhibitor leads to a reduction in TG2 activity, myofibroblast transition and ECM deposition of TG2, FN and activated TGFβ1. Furthermore, TG2 inhibition prevented the increase in FN expression and importantly prevented the increase in expression of the key myofibroblast marker αSMA, whose expression could also be inhibited by the cell impermeable TG2 inhibitor 281 confirming TG2′s action on TGFβ1 is occurring outside the cell. Moreover, TG2 inhibition prevented the increase in FN and αSMA expression that was induced by over-expression of TG2 in NHLFs both in the presence and absence of TGFβ1. These data further support our hypothesis that inhibition of TG2 using either a cell permeable or cell impermeable inhibitor prevents the positive-feedback loop between TG2 and TGFβ1, leading to reduced myofibroblast presence and excessive deposition of ECM proteins.

Previous research showed that TGFβ stimulation of healthy lung fibroblasts leads to an increase in ECM and cell surface levels of TG2 but no change in cytosol TG2 was observed following stimulation (Olsen et al. 2011). In contrast, we found that TG2 protein levels were significantly increased in both whole cell lysates and in the ECM following treatment with TGFβ1 which may be due to the different concentrations of TGFβ1 used in the two studies.

**Fig. 8:**
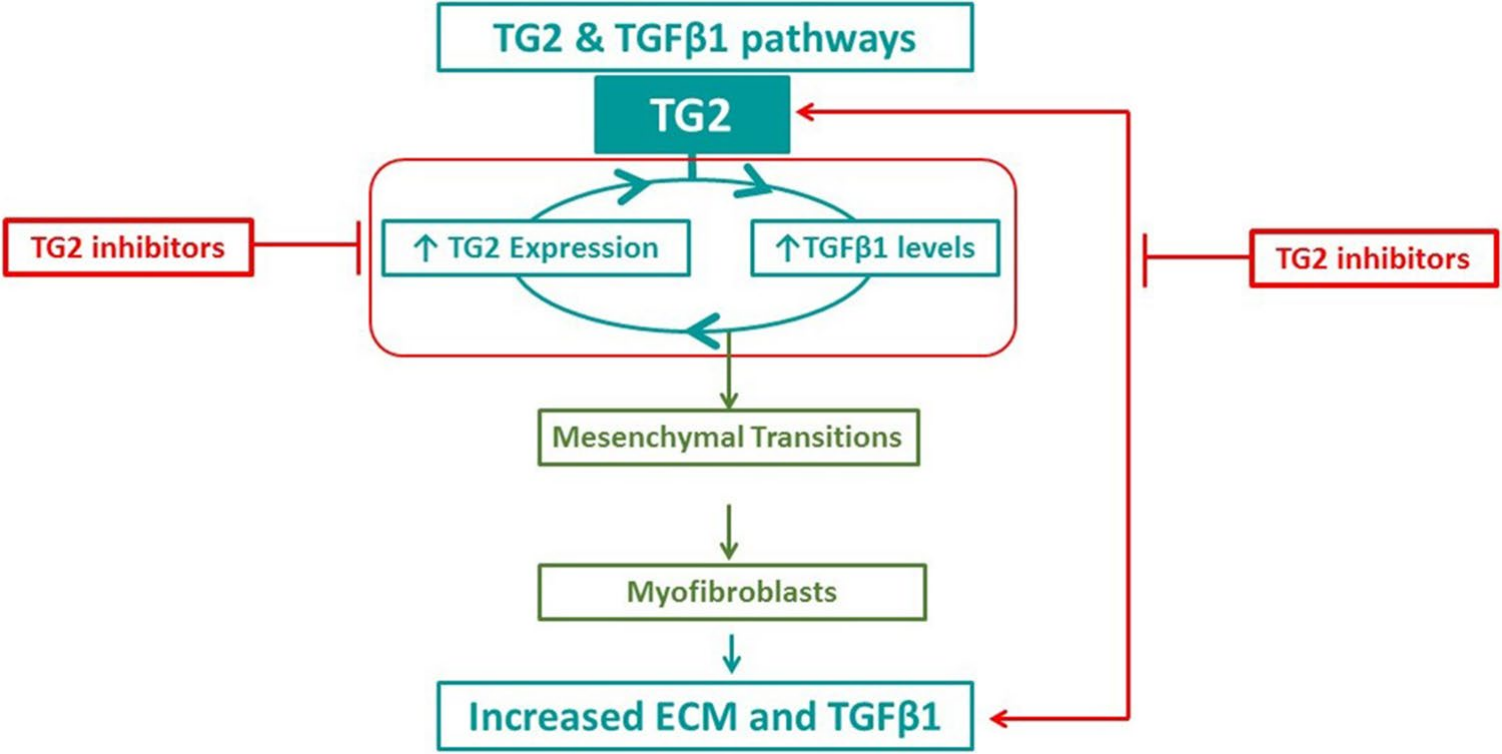
Schematic showing how TG2 inhibitors block fibrosis during IPF. The vicious cycle between TG2 and TGFβ1 leads to the transformation of lung fibroblasts into myofibroblasts which subsequently results in the increase of fibrotic tissue and further active TGFβ1, leading to sustained expression of TG2. Inhibiting this vicious cycle using selective TG2 inhibitors attenuates the progression of fibrosis during IPF

In conclusion, using active site-directed inhibitors that are selective for TG2, we demonstrate that this multifunctional enzyme is critical for maintenance of the myofibroblast phenotype in primary IPF fibroblasts. We show that extracellular TG2 is an important regulator of TGFβ1 activity and TGFβ1-induced lung myofibroblast formation which leads to a vicious cycle involving increased TGFβ activation, increased expression and extracellular activity of TG2 and increased ECM deposition (Fig. 8). Importantly, this study confirms the importance of TG2 as promising therapeutic target for the treatment of IPF.

## Materials and methods

### Chemicals

General chemicals used within this study were purchased from Sigma-Aldrich (UK) unless stated otherwise.

### Cells

Primary NHLFs and Human IPF Fibroblasts cells were supplied by GSK (UK). The cells were used up to a maximum of passage 8. MLECs were gifted by Professor Daniel Rifkin (New York University, USA).

### Cell culture

NHLFs and IPF fibroblastss were cultured in complete DMEM containing Ultraglutamine (glutamine 4.5 g/l), 1% (v/v) non-essential amino acids (NEAA) and 10% (v/v) foetal calf serum (FCS) supplementation. MLECs were cultured in complete DMEM containing 2 mM glutamine, 10% (v/v) FCS and 250 μg/ml G418 sulphate. The medium was changed every 48 to 72 h. NHLFs and IPF fibroblasts were maintained at 37 °C in a 10% CO_2_-humidified incubator, whilst MLECs were cultured at 37 °C in a 5% CO_2_-humidified incubator.

### Cell treatments

TG2-selective inhibitor 1–155 (Badarau et al. 2015) and extracellular-acting TG2 inhibitor R281 (Badarau et al. 2013) were synthesised in Aston University laboratories. Recombinant human TG2 (Rh-TG2) was from Zedira (Germany). Transforming growth factor β1 (TGFβ1) and ERK inhibitor (PD98059) were purchased from R and D Systems (UK).

### Lentiviral transduction of NHLFs

NHLFs were transduced with lentiviral particles containing TG2 and a control empty lentivirus. Lentiviral transduction was performed as previously described (Wang et al. 2013). Cells were cultured to 80% confluency in a 60 mm Petri dish, followed by two rounds of lentiviral transduction. The transduction efficiency was confirmed using Western blotting.

### CRISPR-Cas9 gene editing

The gRNAs were designed using a free online tool (Deskgen). The two gRNA sequences used for TG2 KO were 'GTCTTGCTGGTCCACCACGG' and 'TGAGGCGATACAGGCCGATG' which both target exon 3 in the TGM2 gene. For SMAD3 gene deletion the cells used in this study and the gRNA sequence used to achieve gene editing of Smad3 was ‘GAGCTGACACGGAGACACAT’ as published by Martufi et al. with only a single RNA electroporation performed using the method described in the same paper (Martufi et al. 2019). Briefly, the gRNA was diluted in nuclease-free duplex buffer at 25 µM before boiling at 95 °C for 5 min and then cooling for a further 10 min at RT. The RNA (72.5 pmol) was then complexed with the Cas9 enzyme (60 pmol) and incubated at RT for 10 min before adding 60 pmol of electroporator enhancer and incubating for a further 5 min at RT. IPF fibroblasts (2.5 × 10^5^ cells) were then washed with PBS, pH 7.4, before being pelleted by centrifugation at 90 × g for 10 min. The cells were re-suspended in 15.5 µl P3 solution (Lonza, UK) and then 4.5 µl of the gRNA complex was added. The electroporation was performed in 16-well Nucleocuvette strips. The IPF fibroblasts were then plated into a 24-well plate and incubated in a 37 °C, 10% CO_2_-humidified incubator for 24 h. The next-generation sequencing and analysis was performed by Matteo Martufi (GlaxoSmithKline).

### MLEC TGFβ reporter assay

Active TGFβ levels were measured using the MLEC TGFβ reporter assay (Abe et al. 1994). Fibroblasts were seeded at a density of 5 × 10^4^ cells/well in a 96-well plate in DMEM containing 0.4% FCS. Rh-TG2 was immediately added to the appropriate wells and then the plate was incubated at 37 °C in a 10% CO_2_-humidified incubator for 16–24 h. Following the incubation, MLECs were seeded at a density of 2.5 × 10^4^ cells/well in a collagen-coated 96-well plate using DMEM containing 0.4% FCS and G418 sulphate (250 µg/ml). The MLECs were incubated at 37 °C in a 5% CO_2_-humidified incubator for 3 h before replacing the medium with medium taken from the fibroblasts. The MLECs were cultured overnight with medium from fibroblasts at 37 °C in a 5% CO_2_-humidified incubator before replacing the medium with serum-free medium. Bright-Glo reagent (Promega) was then added to the serum-free medium and the plate was incubated for 2 min before transferring the total volume to a white-walled 96-well plate. The plate was read on a SpectraMax-L luminescent plate reader.

### In situ TG2 activity using FITC-cadaverine incorporation

In situ TG2 activity assay was performed as described previously (Jones et al. 2013). Fibroblasts were seeded at a density of 8 × 10^4^ cells/well in an 8-well chamber slide and incubated overnight in a 37 °C, 10% CO_2_-humidified incubator. Fresh medium and treatments were then added before replacing these after 24 h. Following 48-h incubation with treatments, fresh medium containing 0.5 mM FITC-cadaverine was added and incubated for further 16 h in a 37 °C, 5 or 10% CO_2_-humidified incubator. Following the incubation, medium was discarded and the cells were washed three times with PBS, pH 7.4. The cells were fixed with 3.7% (v/v) paraformaldehyde in PBS, pH 7.4, for 15 min at RT before washing three times with PBS, pH 7.4. The slide was mounted with Vectorshield mounting medium and detected using confocal microscopy. Quantification of fluorescence was performed using ImageJ software.

### Western blotting

Cell lysates were collected in cell lysis buffer [50 mM Tris–HCl, pH 7.4, containing 1% (v/v) nonidet, 0.5% (w/v) Sodium deoxycholate, 0.1% SDS, 1 mM NaF, 1 mM Na_3_VO_4_, 2 mM EDTA, 0.1 mM PMSF (in methanol) and 1% protease inhibitor cocktail] (Wang et al. 2010). Protein concentration was calculated using a *DC* Protein Assay kit (Bio-Rad, UK). Proteins were separated using reducing SDS-PAGE and transferred to a nitrocellulose membrane. The membranes were immuno-probed with protein-specific antibodies (Table 1). The Western blotting protocol was performed as described previously (Wang et al. 2011, 2012). The membrane was stripped and re-probed for GAPDH as an equal loading control that was used to normalize the signal for the target protein in cell lysates.

**Table 1:** All Western blotting and immunocytochemistry antibodies

Primary antibody target	Source
TG2 (Cub7402)	Abcam, UK
FN (F3648**)**	Sigma-Aldrich, UK
αSMA (A2547)	Sigma-Aldrich, UK
p-ERK1/2 (sc-7383 HRP)	Santa Cruz Biotechnology, USA
ERK1/2 (sc-514302 HRP)	Santa Cruz Biotechnology, USA
p-SMAD3 (ab52903)	Abcam, UK
SMAD3 (ab40854)	Abcam, UK
Syndecan-4 (ab24511)	Santa Cruz Biotechnology, USA
GAPDH (ab8245)	Abcam, UK
TGFβ1 (sc-130348 HRP)	Santa Cruz Biotechnology, USA
Collagen type 1 (C2456)	Sigma-Aldrich, UK
Secondary antibody	Source
Swine anti-rabbit HRP-conjugated	Dako, Denmark
Goat anti-mouse HRP-conjugated	Dako, Denmark

For matrix protein analysis where no suitable loading standard was available, equal cell numbers were always used at the start of the experiment. To obtain the cell matrix proteins, gentle removal of cells was undertaken using 5 mM EDTA in PBS, pH74, and the remaining matrix proteins dissolved via scraping into equal volumes of 2 × Laemli buffer (30 µl). The total matrix from each sample was then loaded onto SDS PAGE gels. The western blotting protocol was undertaken as described previously (Wang et al. 2011, 2012). Parallel experiments on the analysis of viable cell numbers with or without treatment with TGF β1 for 72 h indicated that the viable cell numbers were comparable in the TGFβ1 treated and untreated cells (data not shown).

Following primary and secondary antibody incubation, images were captured by a chemiluminescent image analyser Syngene G-box F3 (Cambridge, UK). Densitometry was performed with ImageJ software. The intensity of the bands was normalized against the GAPDH band. The ratios are shown against controls (normalized as 1.0). Data are expressed as mean ratios ± SEM taken from at least three separate experiments unless stated.

### Cell surface TG2 protein detection via biotinylation

Cells were seeded at a density of 5 × 10^5^ cells/60 mm Petri dish and incubated for 2 h before treatments were added. The cells were then incubated for 24 h at 37 °C in a − 10% CO_2_-humidified incubator. The presence of TG2 at the cell surface was detected via biotinylation (Wang et al. 2012) with EZ-link Sulfo-NHS-Biotin (Thermo, UK) according to the manufacturer’s protocol and analysed via Western blotting.

### Isolation of ECM fractions

ECM fractions of the cell cultures were collected as described previously (Wang et al. 2018). Cells were seeded in 35 mm Petri dishes at a density of 2 × 10^5^ cells/dish. Treatments were performed for 72 h before ECM fractions were collected. Cell detachment was performed with 5 mM EDTA in PBS, pH 7.4. The dish was then washed twice with PBS, pH 7.4. The ECM was collected by dissolution in 2 × Laemmli buffer and then underwent protein analysis using Western blotting.

### Statistical analysis

Results shown are mean values ± SEM with data taken from at least three separate experiments unless otherwise stated. The statistical significance between data groups was calculated using either a two-tailed t test or one-way ANOVA using GraphPad Prism. Following analysis using ANOVA, a Tukey post hoc test was performed to identify statistical differences between groups. *p* < 0.05 was considered statistically significant. The results were analysed in Microsoft Excel. GraphPad Prism was used to create graphs, perform statistical analysis and calculate EC50 values.
